# Impact of on-site clinical genetics consultations on diagnostic rate in children and young adults with autism spectrum disorder

**DOI:** 10.1186/s13229-019-0284-2

**Published:** 2019-08-07

**Authors:** Arnold Munnich, Caroline Demily, Lisa Frugère, Charlyne Duwime, Valérie Malan, Giulia Barcia, Céline Vidal, Emeline Throo, Claude Besmond, Laurence Hubert, Gilles Roland-Manuel, Jean-Pierre Malen, Mélanie Ferreri, Sylvain Hanein, Jean-Christophe Thalabard, Nathalie Boddaert, Moïse Assouline

**Affiliations:** 1 Fédération de Génétique Médicale, Institute Imagine, Inserm, Université Paris-Descartes, Hôpital Necker Enfants-Malades, Fondation Elan Retrouvé, Paris, France; 2 Centre de Reference Maladies Rares GénoPsy, Centre Hospitalier le Vinatier, Institut Marc Jeannerod, Bron, France; 3 Hôpital Necker Enfants-Malades, Fondation Elan Retrouvé, Paris, France; 4 Fédération de Génétique Médicale, Institute Imagine, Inserm, Université Paris-Descartes, Hôpital Necker Enfants-Malades, Paris, France; 5 Institute Imagine, Inserm, Université Paris-Descartes, Hôpital Necker Enfants-Malades, Fondation Elan Retrouvé, Paris, France; 6 Institute Imagine, Inserm, Université Paris-Descartes, Hôpital Necker Enfants-Malades, Paris, France; 7 Fondation Elan Retrouvé, Paris, France; 8 MAP5, CNRS, Université Paris-Descartes, Paris, France; 9 Department of Pediatric Radiology, Institute Imagine, Inserm, Université Paris-Descartes, Hôpital Necker Enfants-Malades, Paris, France

**Keywords:** Autism spectrum disorder, Gene panel, Next-generation sequencing, Microarray, Copy number variant, Sequence variant, Fragile X syndrome, Genetic counseling, Genetic diagnosis

## Abstract

**Background:**

Neurogenetics investigations and diagnostic yield in patients with autism spectrum disorder (ASD) have significantly improved over the last few years. Yet, many patients still fail to be systematically investigated.

**Methods:**

To improve access to services, an ambulatory team has been established since 1998, delivering on-site clinical genetics consultations and gradually upgrading services to 502 children and young adults with ASD in their standard environment across 26 day-care hospitals and specialized institutions within the Greater Paris region. The evaluation included a clinical genetics consultation, screening for fragile X syndrome, metabolic workup, chromosomal microarray analysis, and, in a proportion of patients, next-generation sequencing of genes reported in ASD and other neurodevelopmental disorders.

**Results:**

Fragile X syndrome and pathogenic copy number variants (CNVs) accounted for the disease in 10% of cases, including 4/312 (1.3%) with fragile X syndrome and 34/388 (8.8%) with pathogenic CNVs (19 de novo and 4 inherited). Importantly, adding high-throughput resequencing of reported intellectual disability/ASD genes to the screening procedure had a major impact on diagnostic yield in the 141 patients examined most recently. Pathogenic or likely pathogenic sequence variants in 27 disease genes were identified in 33/141 patients (23.4%; 23 were de novo and 10 inherited, including five X-linked and five recessive compound heterozygous variants). Diagnosed cases presented atypical and/or syndromic ASD with moderate to severe intellectual disability. The diagnostic yield of fragile X syndrome and array CGH testing combined with next-generation sequencing was significantly higher than fragile X syndrome and array CGH alone (*p* value 0.009). No inborn errors of metabolism were detected with the metabolic screening.

**Conclusion:**

Based on the diagnostic rate observed in this cohort, we suggest that a stepwise procedure be considered, first screening pathogenic CNVs and a limited number of disease genes in a much larger number of patients, especially those with syndromic ASD and intellectual disability.

**Electronic supplementary material:**

The online version of this article (10.1186/s13229-019-0284-2) contains supplementary material, which is available to authorized users.

## Background

Autism spectrum disorder (ASD) is a major health care issue, affecting 1/200 live births, with a male to female ratio of 4/1 [1, 2]. In the last few years, important advances in deciphering the neurogenetic bases of ASD have been achieved [1, 2]. However, many patients still fail to be offered systematic investigations. In order to improve patients’ access to services, disseminate knowledge, and counteract the loss of opportunity to diagnose a genetic condition, an ambulatory team was established and has visited day-care hospitals across the Greater Paris region since 1998. The team offered comprehensive clinical genetics consultations and gradually improved genetics services to ASD patients in their standard environment.

Here, we show that high-throughput resequencing of reported disease genes had a major impact on diagnostic yield. As cost and access to genomic facilities are common issues, we suggest that a stepwise procedure be considered, first screening a limited number of disease genes in a much larger number of individuals, especially those with syndromic ASD and intellectual disability. Moreover, owing to constraints imposed by the special needs of those patients, we suggest that this flexible method of on-site genetics services be considered, to implement improved standard of care, navigate referrals, and counteract the loss of opportunity to diagnose a genetic condition in patients with ASD and their relatives.

## Patients and methods

The ambulatory team is based in the medical genetics clinic of the Necker-Enfants Malades University hospital, in Paris. Initially, it included one clinical geneticist (0.2 full-time equivalent, FTE), one case manager (0.5 FTE), and one clinical psychologist (0.5 FTE). The team has grown over time and now includes six members: two clinical geneticists (1.4 FTE), two FTE case managers, one FTE genetic counselor, and one FTE neuropsychologist. The institutions that were visited include 26 day-care hospitals and special schooling medical institutions, under the authority of the Greater Paris Regional Health Agency. Initiated in 1998, the program is still ongoing and will continue to serve the community with a multi-annual budget of the Greater Paris Regional Health Agency.

All institutions were visited upon request. They were founded in the mid-1960s at the initiative of parents and family support groups eager to prevent the psychiatric hospitalization of children. Parents’ consent, and when possible, patient’s consent, was obtained prior to the consultation in accordance with French legislation. All patients diagnosed with ASD based on Diagnostic and Statistical Manual of Mental Disorders criteria [3] were offered a consultation. Standardized clinical assessment supported multidimensional symptoms (Childhood Autism Rating Scale, Autism Diagnostic Observation Schedule, and/or Autism Diagnostic Interview-Revised). Global cognitive testing indicated cognitive dysfunctions (especially attention and/or visual-spatial impairments) and various degrees of intellectual disability in all patients. Local child psychiatrists attended the medical genetics consultation. For the sake of privacy, a confidential consultation with the clinical geneticist was offered to the family and seldom accepted. Consultations reviewed personal and family history, pedigree, and photo albums and included a complete clinical examination of the child in the presence of a local team member. The procedure addressed the following questions: (i) is ASD isolated or part of a recognizable syndrome? (ii) is the case sporadic or familial? (iii) with or without intellectual disability? and (iv) are there risk factors? (paternal age, in vitro fertilization, prematurity, drug intake during pregnancy). Owing to rapid changes in the field, a minority of medical genetics records were considered up-to-date. None of these patients has been reported previously.

Ambulatory workup included (i) screening for *FMR1* expansion, (ii) metabolic workup (amino acid and organic acid chromatography, succinyl purines, sialo transferrin, creatine synthesis intermediates), and (iii) array comparative genomic hybridization (CGH), replacing high-resolution karyotype from 2005 onwards [4]. Agilent 60 K microarrays (Agilent Technologies, Santa Clara, CA) were used for genomic copy number analyses on blood samples. Chromosomal rearrangements were confirmed by fluorescence in situ hybridization (FISH). The pathogenicity of copy number variants (CNVs) was assessed according to the guidelines of the American College of Medical Genetics [5]. When this first series of tests were negative, they were followed by a brain magnetic resonance imaging (MRI) in 347/502 patients, with nuclear magnetic resonance (NMR) spectroscopy and a computerized tomography (CT) scan upon short sedation, and electroencephalography (EEG).

From 2014, high-throughput next-generation sequencing (NGS) of intellectual disability/ASD genes was performed in a proportion of families (both parents and the child) [6]. The NGS panel used in this study was an in-house, non-commercial service panel designed at the Imagine Institute in Paris, and was based on a sequence capture method (Agilent Technologies). It screened a total of 439 genes, known to be implicated in intellectual disability/ASD or candidate genes reported at least twice in two distinct studies (Additional file 1: Table S1). Genomic DNA was extracted from peripheral blood using standard procedures. Agilent Sure Select libraries were prepared from 2 μg of genomic DNA sheared with a Covaris S2 Ultrasonicator. Regions of interest were captured with the corresponding 120-bp cRNA baits using the SureSelectXT Target Enrichment Reagent (Agilent) and the Ovation® Target Capture Module (NuGen). The targeted region was sequenced on an Illumina HiSeq2500 (Illumina Inc., San Diego, CA) generating 2 × 130 paired-end reads. Paired-end sequence datasets from Illumina HiSeq2500 runs were treated following three main steps: alignment against human genome release hg19 (using Bwa), calling of single nucleotide variants and small indels (using SAMtools, GATK, and Varscan), and variant annotation based on Ensembl human database (GRCh37 release). Data was integrated in pipelines enabling a CNV analysis based on a double normalization of depth coverage.

A minority of patients benefited from different gene panels or whole exome sequencing as part of research projects. Sequence variants were classified according to the American College of Medical Genetics and Genomics (ACMG) guidelines [7]. Variants were confirmed by Sanger sequencing and segregation analysis was performed in the families for which parental DNA was available. Paternity and maternity were confirmed in all patients carrying a de novo variant. Results and conclusions were communicated to patients and families during subsequent on-site multi-disciplinary consultations. Variants of uncertain significance (VOUS) were not reported to the parents [8–11].

Statistical analyses were performed using the non-parametric Fisher’s exact test (null hypothesis: no percentage difference between the pre-NGS versus NGS tested patients; two-sided test) [12–14].

## Results

A total of 502 patients from 26 institutions were included in the program. There were 351 males and 151 females; most patients were unrelated, except for nine families with two affected siblings, and one family with three affected siblings. The distribution of patients by age categories was as follows: < 10 years, 34; 11–20 years, 194; 21–30 years, 211; > 30 years, 63. The majority of parents were positive about the on-site medical genetics consultations. Less than 1% of families declined the offer to participate, arguing that no immediate benefit would follow. Meeting with families and drawing the pedigree occasionally recognized X-linked or autosomal recessive forms of ASD. Frequently, significant and previously unreported information was first disclosed to the clinical geneticist (family history, affected relatives, serious medical events during pregnancy, in vitro fertilization, etc.). Brain MRI detected overt, yet nonspecific, isolated, or combined anomalies in 146/347 (42%) patients [15], including (i) punctuated white matter hyper intensities, (ii) impaired gray/white matter differentiation of temporal horns, and (iii) dilation of the Virchow-Robin spaces (Table 1).

**Table 1:** Brain MRI anomalies in 146 patients with ASD in day-care hospitals of the Greater Paris region

Brain MRI anomalies	Number of patients
Temporal pole anomalies on T2-weighted images (subcortical hyper-intensity^52^, hypoplasia^2, 37^, dedifferentiation)	36 (24.7%)
White matter hyper-intensities on T2-weighted images (hemispheres^60^, periventricular, insula, pallidum, cerebellum; focal, punctuate, heterotopia)	26 (17.8%)
Cerebellar anomalies (vermian or hemispheric atrophy^37, 54, 67^, hypoplasia^20^, signal anomalies)	25 (17.1%)
Abnormal ventricles (dysmorphism^45^, dilatation^18^, asymmetry)	23 (15.8%)
Corpus callosum anomalies (short, thin^18^, thick^26, 37^, dysmorphic)	19 (13.0%)
Cysts^47^, tumors (teratoms, gangliomas, germinomas)	14 (9.6%)
Dilation of Virchow-Robin spaces^47^	12 (8.2%)
Abnormal pituitary gland^14, 52^	8 (5.5%)
Abnormal gyration (heterotopia, polymicrogyria, pachygyria)	6 (4.1%)

The numbers in superscript refer to the patients listed in Tables 2 and 3

On-site visits allowed the review of laboratory investigations including screening for fragile X syndrome, metabolic workup, and cytogenetic analyses. No screening for fragile X syndrome was available in 312/502 patients. The previously untested patients were screened and 1.3% (4/312) were found to be positive for fragile X syndrome. Systematic metabolic workup made a marginal contribution, especially as neonatal screening for phenylketonuria, and hypothyroidism is widespread in France.

Systematic array CGH replaced high-resolution karyotype from 2005 onwards and was performed in 388/502 patients. Pathogenic CNVs were found in 8.8% (34/388) of cases, including 19 de novo, 4 inherited, and 11 of unknown inheritance (adopted child, parent deceased, or unavailable) (Table 2). Most diagnosed patients presented atypical and/or syndromic ASD with moderate to severe intellectual disability.

**Table 2:** Pathogenic CNVs identified in patients with ASD in day-care hospitals of the Greater Paris region

Patient	Region	Coordinates (GRCh37/hg19)	Del/Dup	Phenotype (MIM number)	Size	Inheritance	Sex
1	1p21.3	(98134258x2,98186019_99530585x1,99612872x2)	Deletion	–	1.4 Mb	NA	M
2	1p36.33p36.32	(0852803_2723463)x1 dn	Deletion	Chromosome 1p36 deletion syndrome (# 607872)	1.9 Mb	De novo	F
3	2p16.3	(50597116_50837494)x1	Deletion	Chromosome 2p16.3 deletion syndrome (*NRXN1* gene) (# 614332)	240 kb	NA	M
4	2p16.3	(508925906x2,50937444_51446873x1,51510902x2)pat	Deletion	Chromosome 2p16.3 deletion syndrome (*NRXN1* gene) (# 614332)	250 kb	Inherited from the father	M
5	4q31.1	(139993209x2,140046328_140323064x1,14037951x2)dn	Deletion	*–*	276 kb	De novo	F
6	5q13.3q14.1	(76116577_78831700)x1 dn	Deletion	–	2.7 Mb	De novo	M
7	6q22.1q22.31	(117955439x2,117998538_123380719x1,123539625x2)dn	Deletion	–	5.4 Mb	De novo	F
8	7q31.1	(113824704_114008914)x1	Deletion	Speech-language disorder-1 (*FOXP2* gene) (# 602081)	184 kb	NA	M
9	8q12.3	(63847208_65755563)x1 dn	Deletion	–	1.9 Mb	De novo	M
10	10q11.22q11.23	(48533668x2,49390457_52415071x1,52566354x2)dn	Deletion	–	3 Mb	De novo	M
11	16p11.2	(28543104_29133735)x1 pat	Deletion	Chromosome 16p11.2 deletion syndrome (*SH2B1* gene) (# 613444)	592 kb	Inherited from the father	M
12	16p13.3	(3776852x2,3831263_3831322x1,3855608x2)	Intragenic deletion in *CREBBP*	Rubinstein-Taybi deletion syndrome (# 610543)	*–*	NA	F
13	17q21.31	(43717703_44210822)x1	Deletion	Koolen-De Vries syndrome (# 610443)	500 kb	De novo	F
14	18q21.33q23	(60610554_77945325)x1	Deletion	Chromosome 18q deletion syndrome (# 601808)	17.3 Mb	NA	M
15	19q12q13.3	Karyotype and FISH analysis (probe YAC 954B2 [provided by Human Polymorphism study Center], location 19q12; locus AFM150xa9)	Deletion	–	–	De novo	M
16	20q11.23q12	(37467951_39961785)x1	Deletion	–	2.5 Mb	NA	F
17	22q11.2	Karyotype and FISH analysis (probes RP11-316 L10 and RP11-1107 K6, location 22q11.2, locus *TBX1*)	Deletion	Velocardiofacial syndrome (# 192430)	–	NA	M
18	22q13.3	Karyotype and FISH analysis (cosmid probe c106G1220P, location 22q13.3, locus *SHANK3*)	Deletion	Phelan-McDermid syndrome (# 606232)	–	De novo	F
19	22q13.33	(51121514x2,51122452_51178264x1,51181762x2)dn	Deletion	Phelan-McDermid syndrome (# 606232)	55.8–60.2 kb	De novo	M
20	Xp11.4	(41510822_41912496)x1 dn	Deletion	Mental retardation and microcephaly with pontine and cerebellar hypoplasia (*CASK* gene) (# 300749)	405 kb	De novo	F
21	1q21.1q21.2	(145747269x2,146324068_149079826x3,149154996x2)dn	Duplication	Chromosome 1q21.1 duplication syndrome (# 612475)	2.7 Mb	De novo	M
22	1q31	Karyotype and FISH analysis (probes RP11-440G22 and RP11-142 L4, location 1q31.2)	Duplication	–	–	NA	F
23	1q32.2	(207780569_208295581)x3	Duplication	–	515 kb	NA	M
24	4p15.3p16.3 4q34.1q35.2	Recombinant chromosome 4 from a pericentric inversion	Duplication Deletion	–	14 Mb 15 Mb	De novo De novo	M
25	5p15.33p14.3	(658561_19955760x3, 20049711x2)dn	Duplication	–	19.3 Mb	De novo	F
26	8p12p11.21	(31396993x2,31488003_43056153x3,43110494x2)dn	Duplication	–	11.6 Mb	De novo	M
27	8q24.13q23	Karyotype and FISH analysis (probe RP11-762A3, location 8q23.3, locus TRPS1 and probe RP11-89P19, location 8q24.1, locus EXT1)	Duplication	–	–	De novo	M
28	14q31.3qter	(88212824_107258824)x3[0.2]dn	Duplication	Mosaic chromosome 14q duplication	19 Mb	De novo	M
29	15q11q13	Karyotype and FISH analysis (probe cos368 H, location 15q11.2)	Duplication	Chromosome 15q11q13 duplication syndrome (# 608636)	–	De novo	M
30	16p13.12p12.3	(14780195x2,15048751_16276115x3,16899616x2)mat	Duplication	–	1.2 Mb	Inherited from the mother	M
31	18p11.32p11.31	(198111_3512486)x3	Duplication	–	3.3 Mb	De novo	M
32	22q11.23	(23668074x2,23739437_24988455x3,25119044x2)mat	Duplication	–	1.2 Mb	Inherited from the mother	M
33	22q13.33	(51112766_51137924)X3	Partial duplication of *SHANK3*	–	Exons 1 to 12	NA (deceased father)	F
34	Xp11	Karyotype 45,X [16]/46,X,idic(X)(p11) [9]	Mosaic isodicentric X chromosome	–	–	NA	F

*F* female, *FISH* fluorescence in situ hybridization, *M* male, *NA* not available (adopted child, parent deceased or unavailable)

Of our 502 patients, 193 were seen prior to and 309 after inception of NGS in 2014. Among the 309 patients seen from 2014 onwards, a pathogenic CNV was found in 18/309. Owing to funding restriction, only a fraction of patients without pathogenic CNVs (141/291) had NGS. NGS consisted in either panel resequencing of 439 known intellectual disability/ASD genes or candidate genes (Additional file 1: Table S1) or different gene panels in 116/141 patients, or whole exome sequencing in 25/141. A pathogenic or likely pathogenic variant was identified in 23.4% (33/141) of cases (Table 3). Most variants occurred de novo (23/33, 70%), while X-linked inheritance accounted for 5/33 (15%) cases and compound recessive heterozygosity for 5/33 (15%) cases. A total of 27 different disease genes were found to be mutated in our series. All diagnosed cases were syndromic forms of ASD, with moderate to severe intellectual disability. VOUS were identified in 23.4% (33/141) cases (data not shown) and were not reported to parents.

**Table 3:** Deleterious sequence variants identified in patients with ASD in day-care hospitals of the Greater Paris region

Patient	Method	Gene	Reference sequence	cDNA and protein changes	Zygosity	Mode of inheritance	Sex	ACMG classification^a^	Evidence	Phenotype (MIM number)
35	ASD/ID panel	*ADNP*	NM_015339	c.2499del, p.Val834Serfs*80	Heterozygous	De novo	M	Pathogenic (Ia)	PVS1, PS2, PM2	Helsmoortel van der Aa syndrome (615873)
36	ASD/ID panel	*ADNP*	NM_015339	c.517C>T, p.Arg173*	Heterozygous	De novo	M	Pathogenic (Ia)	PVS1, PS2, PM2	Helsmoortel van der Aa syndrome (615873)
37	ASD/ID panel	*ANKRD11*	NM_013275	c.3542_3543ins23, p.Arg1182Alafs*144	Heterozygous	De novo	M	Pathogenic (Ia)	PVS1, PS2, PM2	KBG syndrome (148050)
38	ASD/ID panel	*ARID1B*	NM_020732.3	c.4110G>A, p.His1339Ilefs*77 (^b^)	Heterozygous	De novo	M	Pathogenic (Ia)	PVS1, PS2, PS1, PM2	Coffin-Siris syndrome 1 (135900)
39	WES	*ATRX*	NM_000489.3	c.6740A>C, p.His2247Pro	Hemizygous	Inherited from heterozygous mother	M	Likely pathogenic (II)	PS1, PM2, PP2, PP3, PP4	Mental retardation-hypotonic facies syndrome, X-linked (309580)
40	WES	*CACNA1E*	NM_000721.3	c.4688A>G, p.Lys1563Arg	Heterozygous	De novo	M	Likely pathogenic (II)	PS2, PM2, PP2, PP3	Epileptic encephalopathy, early infantile, 69 (618285)
41	WES	*CHD2*	NM_001271.3	c.2352+1G>A, p.Lys730Asnfs*4 Skip of exon 18	Heterozygous	De novo	M	Pathogenic (Ia)	PVS1, PS2, PM2	Epileptic encephalopathy, childhood-onset (615369)
42	WES	*COG4*	NM_015386.2	c.15G>A, p.Met5Ile	Homozygous	Inherited from heterozygous parents	M	Likely pathogenic (V)	PM2, PM3, PP2, PP3, PP4	Congenital disorder of glycosylation, type IIj (613489)
43	WES	*FOXP1*	NM_032682.5	c.1541G>A, p.Arg514His	Heterozygous	De novo	F	Likely pathogenic (II)	PS2, PM2, PP2, PP3	Mental retardation with language impairment and with or without autistic features (613670)
44	ASD/ID panel	*FOXP1*	NM_032682.5	c.1541G>A, p.Arg514His	Heterozygous	De novo	F	Likely pathogenic (II)	PS2, PM2, PP2, PP3	Mental retardation with language impairment and with or without autistic features (613670)
45	WES	*GNAO1*	NM_020988.2	c.736G>A, p.Glu246Lys	Heterozygous	De novo	F	Pathogenic (II)	PS2, PS1, PM2, PP2, PP3, PP4	Epileptic encephalopathy, early infantile 17 (615473)
46^c^	ASD/ID panel	*GRIA3*	NM_000828	c.504del, p.Glu168Aspfs*21	Hemizygous	Inherited from mother with somatic mosaicism	M	Pathogenic (Ib)	PVS1, PM2, PP1-M	Mental retardation, X-linked 94 (300699)
47^c^	ASD/ID panel	*GRIA3*	NM_000828	c.504del, p.Glu168Aspfs*21	Hemizygous	Inherited from mother with somatic mosaicism	M	Pathogenic (Ib)	PVS1, PM2, PP1-M	Mental retardation, X-linked 94 (300699)
48	ASD/ID panel	*GRIA3*	NM_000828	c.1990C>G, p.Pro664Ala	Hemizygous	Inherited from heterozygous mother	M	Likely pathogenic (II)	PS1, PM2, PP2, PP3, PP4	Mental retardation, X-linked 94 (300699)
49	ASD/ID panel	*GRIN2B*	NM_000834.4	c.2087G>A, p.Arg696His	Heterozygous	De novo	F	Pathogenic (II)	PS2, PS1, PM2, PP2, PP3, PP4	Mental retardation, autosomal dominant 6 (613970)
50	ASD/ID panel	*GRIN2B*	NM_000834.4	c.2084T>C, p.Ile695Thr	Heterozygous	De novo	M	Pathogenic (II)	PS2, PS1, PM2, PP2, PP3, PP4	Mental retardation, autosomal dominant 6 (613970)
51	ASD/ID panel	*HUWE1*	NM_031407.6	c.1736A>C, p.Asn579Thr	Hemizygous	Inherited from heterozygous mother	M	Likely pathogenic (II)	PS1, PM2, PP2, PP3, PP4	Mental retardation, X-linked syndromic (300706)
52	Epilepsy panel	*IQSEC2*	NM_001111125.2	c.2272C>T, p.Arg758*	Heterozygous	de novo	F	Pathogenic (Ia)	PVS1, PS2, PM2	Mental retardation, X-linked 78 (309530)
53	WES	*KCNB1*	NM_004975.2	c.128A>G, p.Glu43Gly	Heterozygous	De novo	M	Likely pathogenic (II)	PS2, PM2, PP3, PP2	Epileptic encephalopathy, early infantile 26 (616056)
54	ASD/ID panel	*KDM6A*	NM_021140.3	c.2944G>T, p.Gly982*	Heterozygous	De novo	M	Pathogenic (Ia)	PVS1, PS2, PM2	Kabuki syndrome 2 (300867)
55	WES	*LINS1*	NM_001040616.2	c.1921del, p.Glu641Serfs*4	Homozygous	Inherited from heterozygous parents	M	Likely pathogenic (V)	PM2, PM3, PP2, PP3, PP4	Mental retardation, autosomal recessive 27 (614340)
56	ASD/ID panel	*MED13L*	NM_015335.4	c.1708_1709del, p.Ser570Phefs*27	Heterozygous	De novo	F	Pathogenic (Ia)	PVS1, PS2, PM2	Mental retardation and distinctive facial features with or without cardiac defects (616789)
57	ASD/ID panel	*MYT1L*	NM_015025.3	c.1579G>C, p.Gly527Arg	Heterozygous	De novo	F	Pathogenic (II)	PS2, PS1, PM2, PP2, PP3, PP4	Mental retardation, autosomal dominant 39 (616521)
58	ASD/ID panel	*NAA10*	NM_003491.3	c.236G>A, p.Arg79His	Heterozygous	De novo	M	Likely pathogenic (II)	PS2, PM2, PP2, PP3	Ogden syndrome (300855)
59	WES	*PHF6*	NM_032458.2	c.385C>T, p.Arg129*	Heterozygous	De novo	F	Pathogenic (Ia)	PVS1, PS2, PM2	Borjeson-Forssman-Lehmann syndrome (301900)
60	WES, Epilepsy panel	*RORB*	NM_006914.3	c.640C>T, p.Arg214*	Heterozygous	De novo	F	Pathogenic (Ia)	PVS1, PS2, PM2	Epilepsy, idiopathic generalized, susceptibility to, 15 (618357)
61	ASD/ID panel	*SHANK3*	NM_033517.1	c.5021G>A, p.Gly1674Asp	Heterozygous	Inherited from the affected mother	M	Likely pathogenic (II)	PP1-S, PM2, PP2, PP3, PP4	Phelan-McDermid syndrome (606232)
62	ASD/ID panel	*SHANK3*	NM_033517.1	c.3679dup, p.(Ala1227Glyfs*69)	Heterozygous	De novo	M	Pathogenic (Ia)	PVS1, PS2, PM2	Phelan-McDermid syndrome (606232)
63	ASD/ID panel	*SLC6A1*	NM_003042	c.752T>C, p.Leu251Pro	Heterozygous	De novo	F	Likely pathogenic (II)	PS2, PM2, PP3, PP2	Myoclonic-atonic epilepsy (616421)
64	Epilepsy panel	*STXBP1*	NM_003165.3	c.87+1G>T, p.?	Heterozygous	De novo	M	Pathogenic (Ia)	PVS1, PS2, PM2	Epileptic encephalopathy, early infantile, 4 (612164)
65	ASD/ID panel	*SZT2*	NM_015284.3	c.1261+1G>A, p.? c.6113A>G, p.Tyr2038Cys	Compound heterozygous	Inherited from heterozygous parents	F	Likely pathogenic (V)	PVS1, PM2, PM3, PP2, PP3, PP4	Epileptic encephalopathy, early infantile 18 (615476)
66	ASD/ID panel	*TLK2*	NM_006852.3	c.1015C>T, p.Arg339Trp	Heterozygous	De novo	M	Pathogenic (II)	PS2, PS1, PM2, PP2, PP3, PP4	Mental retardation, autosomal dominant 57 (618050)
67	WES	*TUSC3*	NM_006765.3	c.787_788insC, p.Asn263Thrfs*	homozygous	Inherited from heterozygous parents	M	Pathogenic (Ib)	PVS1, PM2, PM3, PP2	Mental retardation, autosomal recessive 7 (611093)

*ASD* autism spectrum disorder, *F* female, *ID* intellectual disability, *M* male, *WES* whole exome sequencing

^a^Variants were assessed for pathogenicity according to the American College of Medical Genetics and Genomics (ACMG) criteria [7]

^b^In patient 38, the c.4110G>A variant in *ARID1B* is predicted to result in a synonymous substitution (p.Pro1370=) in the last base pair of exon 17. Further studies indicated that this variant affects the splice donor site and induces skipping of exon 17, causing a frameshift and premature termination (p.His1339Ilefs*77) [16]

^c^Patients 46 and 47 are siblings

Overall, on-site medical genetics consultations in specialized institutions identified previously undiagnosed genetic conditions in 71 ASD children and young adults and the implementation of NGS significantly improved diagnostic yield. The difference in diagnostic yield of array CGH and fragile X syndrome testing either alone or combined with NGS was strongly significant (Fisher’s exact test, *p* value 0.00998).

Parents frequently mentioned that putting a name on the disease mechanism was not perceived as a “stigmatization,” but instead, they described it as a “relief” that helped them understand and overcome hardships and a connection to family support groups and other families facing similar situations.

Occasionally, couples reported that genetic counseling arrived too late, when they already had a second affected child (or relative) or had given up the idea of having another child. Local team members frequently considered identifying the disease mechanism as an opportunity to improve specific management and gain access to relevant literature and future clinical trials. When the procedure proved unsuccessful, on-site follow-up appointments were offered to families and possible inclusion in research programs was discussed (whole exome and whole genome sequencing).

## Discussion

Taking advantage of on-site medical genetics consultations, we estimated the impact of systematic resequencing of reported disease genes on the diagnostic rate in day-care hospitals and special schooling institutions within the Greater Paris area. While array CGH and screening for fragile X syndrome detected pathogenic variants in 10% of patients, further implementation of high-throughput sequencing of intellectual disability/ASD genes identified pathogenic or likely pathogenic variants in 23.5% of investigated patients. Most variants occurred de novo and only 27 genes were found to be mutated in our series [7, 8]. All diagnosed cases were syndromic forms of ASD, with moderate to severe intellectual disability. Some patients had undiagnosed early-onset, transient epilepsy, later ascribed to a genetic condition when deferred behavioral problems occurred. Overall, on-site medical genetics consultations identified previously undiagnosed genetic conditions in 71 ASD children and young adults. This diagnostic yield may be an under-estimate, given that variants of uncertain significance and variants in strong candidate genes were not regarded as the cause of the disease. With the rapid pace of gene discovery in intellectual disability and ASD, some of these uncertain findings will likely be reclassified as pathogenic over time.

Based on this study, we suggest offering systematic array CGH and panel resequencing of known disease genes in syndromic/atypical ASD individuals with an associated intellectual disability. Moreover, we suggest that a stepwise procedure be considered, first screening a limited number of disease genes in a much larger number of patients, especially those with syndromic ASD and intellectual disability. In the future, current guidelines will hopefully mention genetics screening of the most frequent ASD genes as an explicit recommendation to professionals, which is not currently the case [17, 18].

While recognizing a genetic condition had no immediate impact on the case management, this information was often received by parents as a “relief” that helped them overcome hardship and alleviate the sense of guilt and self-blame of having given birth to a child with ASD. Relating to support groups and other families facing similar situations was also appreciated, as it fostered studies aimed at delineating natural history and the long-term outcome of ASD sub-types. On-site consultations also helped offering actionable recommendations and cognitive/behavioral interventions [19, 20].

Conversely, on-site medical genetics consultations had a significant impact on genetic counseling especially when de novo sequence variants or CNVs were identified, as they significantly reduced recurrence risk to parents and relatives (with the reservation of low-recurrence risk germline mosaicism). Omitting or postponing medical genetics consultations and failing to warn of potential genetic risks may have serious consequences in inherited forms of ASD.

The reason why so many patients failed to be systematically investigated before our on-site consultations remains unclear. Possible explanations include a limited number of clinical experts, the congestion or inadequacy of outpatient hospital consultations for patients with special needs, and a lack of funding for genetics services. Furthermore, while parents usually accept being referred to a child neurologist, the perception of a genetics consultation is a much more sensitive issue at the early stages of the disease, i.e., when parents first meet with a child psychiatrist. In contrast, the opportunity to conduct or update the etiological investigations was accepted more easily later on, when there is no doubt regarding the ASD diagnosis, but there are still many remaining mechanism-related questions.

This study shows that ASD children and young adults admitted in specialized institutions within the Greater Paris area (and probably other regions of France as well) had limited access to genetic advances. Deferred, optional on-site interventions may help by offering specialized consultations and counteracting the loss of opportunity to diagnose a genetic condition for both patients and relatives. The fact that genetics services are underused by affected families is not specific to France; it is a major challenge worldwide [21]. For instance, a Spanish study exploring access to genetics services and parental perception of genetic risk in children with ASD revealed striking underuse of genetics services, with only 30% of families visiting a genetics service and 13% of patients undergoing the recommended genetic tests [22]. Similarly, a recent Taiwanese study revealed that two-thirds of parents with children with ASD had never heard about genetic testing for ASD, while the majority (71%) expressed an interest in learning more about such testing [23]. This lack of service provision significantly impacted family planning in both studies.

### Limitations

It is worth noting that our study has several limitations. First, owing to the number of patients reported here, no details on the clinical assessment (CARS, ADOS, ADI-R) or the level of intellectual disability could be individually provided for patients carrying pathogenic CNVs or sequence variants. Second, patients seen by our ambulatory services in specialized institutions possibly differ from those who visit regular clinics, as the most severe cases are selected over time. It is likely that the less severely affected ones are not referred to the institutions visited, and that in these patients, the etiologic yield of the genetic explorations performed here could be lower. Third, the eponym of ASD actually comprises a variety of conditions, including a significant amount of overlooked genetic conditions (i.e.*,* early-onset transient epileptic encephalopathy). Owing to difficulties accessing long-term medical/medico-social facilities for disabled children, recognition of “autistic features” in a disabled child might have channeled many patients towards these high-quality institutions. Finally, an obvious limitation stems from public funding restriction, as only a fraction of patients without pathogenic CNVs had NGS.

## Conclusions

We suggest that on-site clinical genetics consultations be considered in day-care hospitals and specialized institutions, to implement a standard of care, navigate referrals, and counteract the loss of opportunity to diagnose a genetic condition in ASD patients. Particular attention should be paid to a stepwise procedure, first screening for pathogenic CNVs and sequence variants in frequently mutated genes in a much larger number of children with syndromic ASD and intellectual disability.

## Additional file

Additional file 1: **Table S1.** List of 439 known intellectual disability/ASD genes or candidate genes tested by panel resequencing. (XLSX 196 kb)

## Data Availability

Clinical information, brain MRIs, laboratory results, and panel data are available by mail to nicole.guyot-berard-ext@aphp.fr.

## Abbreviations

Array CGH: Array comparative genomic hybridization
ASD: Autism spectrum disorder
CNV: Copy number variant
CT scan: Computerized tomography scan
EEG: Electroencephalography
*FMR1*: Fragile X mental retardation 1
MRI: Magnetic resonance imaging
NGS: Next-generation sequencing
NMR spectroscopy: Nuclear magnetic resonance spectroscopy
VOUS: Variant of uncertain significance
